# Research Progress and Application Prospects of Solid-State Hydrogen Storage Technology

**DOI:** 10.3390/molecules29081767

**Published:** 2024-04-12

**Authors:** Yaohui Xu, Yang Zhou, Yuting Li, Zhao Ding

**Affiliations:** 1 Laboratory for Functional Materials, School of New Energy Materials and Chemistry, Leshan Normal University, Leshan 614000, China; 2 School of Textile Science and Engineering, State Key Laboratory of New Textile Materials and Advanced Processing Technology, Wuhan Textile University, Wuhan 430200, China; 3 Leshan West Silicon Materials Photovoltaic New Energy Industry Technology Research Institute, Leshan 614000, China; 4 College of Materials Science and Engineering, National Engineering Research Center for Magnesium Alloys, Chongqing University, Chongqing 400044, China; 5 National Innovation Center for Industry-Education Integration of Energy Storage Technology, Chongqing University, Chongqing 400044, China

**Keywords:** solid-state hydrogen storage, hydrogen energy, new energy vehicles, distributed energy, collaborative innovation, carbon neutrality

## Abstract

Solid-state hydrogen storage technology has emerged as a disruptive solution to the “last mile” challenge in large-scale hydrogen energy applications, garnering significant global research attention. This paper systematically reviews the Chinese research progress in solid-state hydrogen storage material systems, thermodynamic mechanisms, and system integration. It also quantitatively assesses the market potential of solid-state hydrogen storage across four major application scenarios: on-board hydrogen storage, hydrogen refueling stations, backup power supplies, and power grid peak shaving. Furthermore, it analyzes the bottlenecks and challenges in industrialization related to key materials, testing standards, and innovation platforms. While acknowledging that the cost and performance of solid-state hydrogen storage are not yet fully competitive, the paper highlights its unique advantages of high safety, energy density, and potentially lower costs, showing promise in new energy vehicles and distributed energy fields. Breakthroughs in new hydrogen storage materials like magnesium-based and vanadium-based materials, coupled with improved standards, specifications, and innovation mechanisms, are expected to propel solid-state hydrogen storage into a mainstream technology within 10–15 years, with a market scale exceeding USD 14.3 billion. To accelerate the leapfrog development of China’s solid-state hydrogen storage industry, increased investment in basic research, focused efforts on key core technologies, and streamlining the industry chain from materials to systems are recommended. This includes addressing challenges in passenger vehicles, commercial vehicles, and hydrogen refueling stations, and building a collaborative innovation ecosystem involving government, industry, academia, research, finance, and intermediary entities to support the achievement of carbon peak and neutrality goals and foster a clean, low-carbon, safe, and efficient modern energy system.

## 1. Introductions

Energy is the material basis for human society’s survival and development. While the large-scale development and utilization of fossil energy have promoted the progress of human civilization, they have also led to increasingly severe problems regarding resource depletion and environmental pollution [1,2,3]. Vigorously developing renewable and clean energy and achieving low-carbon or even zero-carbon development have become essential paths for the global energy transition [4]. Hydrogen energy, as an ideal secondary energy carrier, is attracting increasing attention from various sectors. Hydrogen is the simplest and most abundant element in the universe. In nature, hydrogen mainly exists in the form of compounds such as water and hydrocarbons. Hydrogen releases a large amount of heat when burned, producing only water with almost no pollutant emissions, making it a true “clean fuel” [5,6]. In addition, hydrogen has the advantages of a high energy density (142 MJ/kg, three times that of gasoline) and wide availability (water, biomass, etc., can all be used to produce hydrogen). Therefore, constructing an energy utilization system with hydrogen as the medium—“hydrogen energy”—is becoming another important energy form after “electric energy” [7,8]. Hydrogen energy can be widely applied in transportation, industry, construction, power, and other fields. In the transportation sector, fuel cell vehicles use hydrogen as “fuel” and generate electricity directly through electrochemical reactions, with an efficiency more than 30% higher than internal combustion engines. They are considered the most promising new energy vehicle type [9]. In the industrial sector, hydrogen can be used as a clean heat source and reducing agent to replace coke and achieve “hydrogen metallurgy”, thereby significantly reducing carbon emissions from high energy-consuming industries such as steel and chemical industries [10,11]. In the construction sector, hydrogen fuel cell combined cooling, heating, and power (CCHP) systems can replace traditional gas boilers and provide stable and environmentally friendly energy for buildings. In the power sector, hydrogen energy generation can be used as a backup power source for renewable energy sources such as wind and solar, and can also form megawatt and gigawatt-level hydrogen energy storage power stations to participate in grid load regulation [12,13,14,15]. In short, hydrogen energy provides a new solution for sustainable development. China attaches great importance to the development of the hydrogen energy industry. In 2019, hydrogen energy was included in the government work report for the first time [16]. In 2021, the State Council issued the “Opinions on Accelerating the Construction of a Unified National Market”, clearly stating that developing hydrogen energy and other industries is an important measure for building a new development pattern [16]. In October of the same year, five ministries and commissions, including the National Development and Reform Commission, jointly issued the “Guiding Opinions on Accelerating the Development of Energy Storage”, listing hydrogen storage as an emerging energy storage technology that needs to be focused on [16].

Driven by various policy incentives, China’s hydrogen energy industry ushered in unprecedented development opportunities [16,17]. According to forecasts by the China Hydrogen Alliance, under the 2030 carbon peak scenario, China’s hydrogen demand will reach 37.15 million tons, accounting for about 5% of the final energy consumption [18]. Among them, industrial decarbonization hydrogen will be 15.9 million tons, hydrogen for fuel cell vehicles in the transportation sector will be 1.3 million tons, and hydrogen for distributed power generation and grid peak shaving will be 2.25 million tons. By 2060, when carbon neutrality is achieved, hydrogen demand is expected to exceed 130 million tons, accounting for about 20% of the final energy consumption. Among them, the hydrogen used in the industrial sector will be as high as 77.94 million tons, 40.51 million tons in the transportation sector, 5.85 million tons in the construction sector, and 6 million tons for power generation and grid peak shaving. Such a huge application prospect is bound to leverage a trillion-level industrial scale. To truly realize the economic and environmental benefits of hydrogen energy, a compatible production, storage, and transportation system must be established.

Among them, storage and transportation, as key links connecting the supply and consumption sides, have a particularly prominent impact on the safety, accessibility, and economy of hydrogen energy. According to statistics, hydrogen storage and transportation costs account for more than 50% of the entire cost of the hydrogen energy supply chain [19,20]. Therefore, vigorously developing efficient storage and transportation technologies is of decisive significance for promoting the sustainable and healthy development of the hydrogen energy industry. At present, there are three main forms of hydrogen storage: gaseous, liquid, and solid-state. Gaseous hydrogen storage is filled at high pressure (35–70 MPa) and can achieve a certain amount of storage, but the energy density is low (40 kg/m^3^@70 MPa) and there are certain safety hazards. Liquid hydrogen storage uses cryogenic liquefaction (−253 °C), and the energy density can reach 70 kg/m^3^, but the energy consumption is high (12 kWh/kg), the cost is high (USD > 5.7/kg), and the evaporation loss of the liquid hydrogen tank is also a problem. Solid-state hydrogen storage uses chemical or physical interactions to reversibly adsorb hydrogen in solid materials. It has a low operating pressure (0.1–5 MPa), high energy density (100–130 kg/m^3^), and good safety. Coupled with its flexible storage methods (block, granular, powder), it is very suitable for mobile applications such as on-board and distributed applications. Therefore, it is considered one of the most promising innovative hydrogen storage technologies for commercial development.

This article will focus on the cutting-edge field of solid-state hydrogen storage. Firstly, it will introduce the basic principles and representative material systems of solid-state hydrogen storage. Then, it will sort out the research and development status of solid-state hydrogen storage devices. After that, it will focus on analyzing their application potential in scenarios such as on-board hydrogen storage and hydrogen refueling stations. Finally, it will look forward to the development prospects of solid-state hydrogen storage technology and put forward some suggestions in order to provide references for the innovation of hydrogen storage technology in China.

## 2. Hydrogen Storage Technologies

### 2.1. Compressed Gaseous Hydrogen Storage

Gaseous hydrogen storage is a method of storing hydrogen using high-pressure containers. According to the pressure level, storage containers can be divided into Type I (<20 MPa), Type II (20–30 MPa), Type III (30–45 MPa), and Type IV (>45 MPa) [21,22]. Type I cylinders are usually made of seamless steel cylinders, and Type II cylinders are wound with glass-fiber-reinforced materials on the outside of the steel cylinders. The 35 MPa hydrogen storage cylinders used in conventional vehicles belong to Type III, and the 70 MPa cylinders used in buses belong to Type IV. Type IV cylinders are mostly made of aluminum liners and are fully wound with carbon fiber structures, which have high strength and a light weight, and are currently the mainstream solution for on-board hydrogen storage. Table 1 summarizes the main technical specifications of the four hydrogen cylinders. Gaseous hydrogen storage is convenient to operate and has a fast filling speed (3–5 min), with good compatibility with existing gas station processes. However, its disadvantages are also obvious: first, the energy density is low, only 40 kg/m^3^ at 70 MPa, which makes it difficult to meet the requirements for a long cruising range; second, there are certain risks regarding leakage and explosion, which place high demands on container materials and valve seals; third, the energy consumption is high, and the filling power consumption of 70 MPa hydrogen storage cylinders is as high as 3–4 kWh/kg [23,24,25]. Therefore, how to further improve the hydrogen storage density while ensuring safety is the key to gaseous hydrogen storage.

For hydrogen storage technology, its cost structure can be divided into three main aspects: equipment costs, operational costs, and safety and regulatory costs. In gaseous hydrogen storage technology, equipment costs mainly include high-pressure gas cylinders and compressors, accounting for approximately 40–60% of the total cost. Because high-pressure hydrogen containers require special materials and processes to withstand high pressures while ensuring safety and durability, compressors are also necessary for hydrogen filling processes. Therefore, equipment costs are the highest proportion for gaseous hydrogen storage technology. In terms of operational costs, the major energy consumption in gaseous hydrogen lies in the electrical energy consumption during the filling process, which accounts for about 20–40% of the total cost, with routine maintenance accounting for approximately 5%. Due to the drawback of potential leakage in high-pressure gaseous hydrogen technology, expenditures for safety and regulatory compliance are needed, accounting for about 5–15% of the total cost. Taking the popular 20 MPa long-tube trailer as an example, costs increase steadily with transport distance, with prices ranging approximately from USD 0.6 to USD 7 per kilogram for transport distances of 50–500 km. To comprehensively reduce the cost of gaseous hydrogen, three aspects need to be addressed: (1) the research and development of new high-performance materials to reduce the weight and cost of containers while improving safety; (2) the optimization of compressor efficiency to reduce energy consumption; and (3) the adoption of advanced monitoring and control technologies to enhance storage system safety while reducing safety costs.

### 2.2. Cryogenic Liquid Hydrogen Storage

Liquid hydrogen storage uses cryogenic liquefaction to store hydrogen. Liquid hydrogen storage employs low-temperature liquefaction and organic liquid substances to store hydrogen. Table 2 summarizes the main technical specifications of two liquid hydrogen storage methods.

At atmospheric pressure, the liquefaction temperature of hydrogen is −253 °C, and the volumetric energy density can reach 70 kg/m^3^, which is nearly twice that of 70 MPa gaseous hydrogen [26]. Liquid hydrogen tanks generally adopt a vacuum multi-layer insulation structure, with a stainless steel liner and a carbon fiber wound layer as the outer shell, and a vacuum insulation interlayer and multi-layer reflective screens between the inner and outer layers [27]. The energy density of liquid hydrogen storage is high, reducing the hydrogen storage space. And the evaporation latent heat of liquid hydrogen is very large, which has advantages in thermal management and safety protection. Companies such as BMW and Toyota have all launched liquid hydrogen fuel cell vehicle prototypes with a cruising range of 500 km. However, liquid hydrogen storage also has problems that cannot be ignored: first, the liquefaction energy consumption is high, with a theoretical power of 12 kWh/kg and an actual power consumption of more than 20 kWh/kg; second, the structure of the liquid hydrogen tank is complex and costly, reaching more than USD 2857.1/kg; third, the evaporation loss is inevitable, with a daily evaporation rate of about 3% [28,29,30,31]. Therefore, reducing energy consumption and cost and improving the insulation performance are the difficulties associated with promoting the large-scale application of liquid hydrogen.

Organic liquid hydrogen storage lies in leveraging the characteristics of organic compounds to adsorb or absorb hydrogen, thereby storing it in a liquid carrier [32,33]. These organic compounds often possess high porosity or polarity, facilitating the efficient adsorption and release of hydrogen. Under the influence of pressure and temperature, hydrogen is adsorbed or absorbed into the organic material, forming hydrogen compounds and enabling its storage [34,35,36]. This method offers several advantages. Firstly, it typically boasts a higher hydrogen storage density compared to other methods, such as compressed or liquid hydrogen, thus providing greater storage capacity. Additionally, it enhances safety during storage and transportation, as hydrogen is adsorbed or absorbed into a solid or liquid carrier, mitigating the risks associated with handling gaseous hydrogen. Moreover, organic liquid hydrogen storage can be conducted under milder conditions compared to alternatives, improving operational convenience and safety. However, this storage method also presents certain drawbacks. Despite its high storage capacity, organic liquid hydrogen storage tends to exhibit lower power density, meaning it stores less hydrogen per unit volume [37,38,39,40]. Furthermore, the adsorption and desorption rates are typically slower, potentially affecting the release rate of hydrogen and its practicality in certain applications. Additionally, some organic compounds may experience periodic deactivation or degradation, compromising their long-term hydrogen storage performance and stability.

In liquid hydrogen storage, equipment costs, including low-temperature liquefaction facilities and cryogenic storage tanks, constitute a relatively high proportion of the total cost, approximately 50–70%. Meanwhile, during operation, significant energy consumption costs, accounting for about 20–30% of the total cost, may arise from the liquefaction cooling demand, with maintenance costs relatively lower at around 5–10%. Considering the safety of low-temperature storage, safety and regulatory expenditures may account for 10–20% of the total cost. Within a transport range of 50–500 km, the transportation price for liquid hydrogen is approximately USD 2–3.1 per kilogram. Cost optimization can be pursued through the following: (1) improving the liquefaction efficiency of hydrogen through technological innovation to reduce energy consumption; (2) developing more efficient insulation materials and tank designs to minimize hydrogen evaporation losses during storage; and (3) developing specialized low-temperature safety technologies and equipment to enhance overall system safety.

### 2.3. Solid-State Hydrogen Storage

#### 2.3.1. Hydrogen Storage Principles

**Figure 1:**
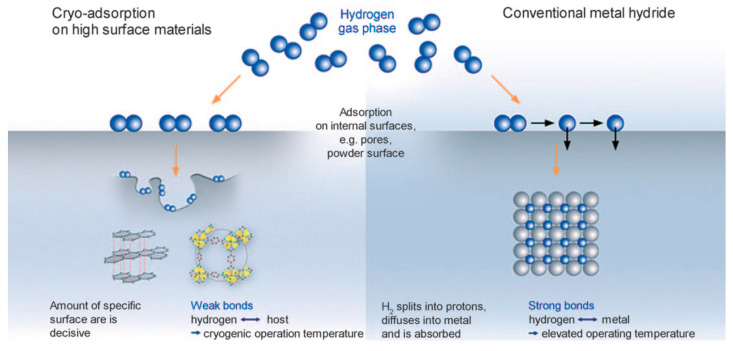
Schematic diagrams illustrating the principles of physical hydrogen storage and chemical hydrogen storage [41].

Solid-state hydrogen storage can be categorized into two main types: physical adsorption and chemical adsorption, as illustrated in Figure 1 [41]. Physical adsorption, also known as physisorption, is a process where gas molecules adhere to a solid surface through van der Waals forces without undergoing any chemical reactions. The adsorption occurs due to the attractive forces between the gas molecules and the surface of the adsorbent material. This type of adsorption is typically reversible and depends on factors such as the surface area, pore size, and affinity between the gas molecules and the adsorbent material. In physical adsorption, the adsorbate molecules are held onto the surface of the adsorbent by weak forces, such as London dispersion forces, dipole–dipole interactions, and hydrogen bonding. As a result, physical adsorption is often characterized by its relatively low heat of adsorption and is highly dependent on temperature and pressure. The adsorption capacity of physical adsorbents can be enhanced by increasing the surface area of the adsorbent material or by optimizing the pore structure to provide more sites for gas molecules to adhere to. The common materials used for physical adsorption include activated carbons, zeolites, and certain metal–organic frameworks (MOFs).

Chemical adsorption, also known as chemisorption, involves the formation of chemical bonds between the adsorbate molecules and the surface of the adsorbent material. Unlike physical adsorption, chemisorption results in the formation of new chemical entities on the surface of the adsorbent, leading to irreversible adsorption behavior. This process typically requires the activation of chemical bonds on the surface of the adsorbent material, often through processes such as oxidation or reduction. Chemisorption is characterized by its higher heat of adsorption compared to physical adsorption, indicating the involvement of stronger chemical bonds in the adsorption process. The adsorption capacity of chemisorbents is determined by the availability of active sites on the adsorbent surface and the strength of the chemical bonds formed between the adsorbate and the adsorbent. Common examples of chemisorption include the adsorption of gases such as hydrogen on metal surfaces, where hydrogen molecules dissociate and form metal hydrides on the surface. Other examples include the adsorption of gases on catalyst surfaces, where chemical reactions between the adsorbate and the surface lead to the formation of new products.

#### 2.3.2. Hydrogen Storage Materials

There are many types of solid-state hydrogen storage materials, mainly including metal hydrides, complex hydrides, chemical hydrides, etc. Among them, metal hydrides have become the most studied and most promising type of application due to their high hydrogen storage capacity and good cyclic stability [42,43,44,45]. According to their chemical composition, metal hydrides can be further divided into AB_5_ type (such as LaNi_5_), AB_2_ type (such as ZrV_2_), AB type (such as TiFe), A_2_B type (such as Mg_2_Ni), etc. They are usually composed of one element with strong hydrogen absorption activity (A) and one element with weak hydrogen absorption activity (B). During dehydrogenation, the two elements work together to regulate the binding force of hydrogen. A is usually selected from rare earth (La, Ce), magnesium (Mg), titanium (Ti), etc., while B is selected from transition metals (V, Cr, Mn, Fe, Co, Ni).

In addition to metal hydrides, complex hydrides are also a type of hydrogen storage material with great application prospects [46,47]. Complex hydrides refer to complex hydrides containing coordinate bonds, such as ammonia borane (NH_3_BH_3_), lithium borohydride (LiBH_4_), etc. They form multi-step reversible dehydrogenation reactions through the interaction between ligands and metal centers, which can achieve high-capacity hydrogen storage, but the reaction enthalpy is large and dehydrogenation usually requires temperatures above 200 °C [48]. In order to improve the dehydrogenation kinetics, transition metals such as Ti and Ce are often introduced as catalysts to increase the reversible hydrogen storage capacity and improve the cyclic stability of the materials.

In addition to chemical hydrogen storage, there exists a class of hydrogen storage materials utilizing physical adsorption, termed physical adsorbent materials. Among these, activated carbon stands out as a conventional and widely employed physical adsorbent material, owing to its porous structure and high specific surface area, rendering it an ideal medium for hydrogen storage. Carbon nanotubes have garnered significant attention due to their unique structure and excellent mechanical properties. Moreover, metal–organic frameworks (MOFs), emerging as a novel material, exhibit an exceptionally high specific surface area (up to 7000 m^2^/g) and tunable pore structures, positioning them as one of the most promising materials in the field of physical adsorption hydrogen storage. The design flexibility of MOFs enables the optimization of hydrogen adsorption performance through structural and compositional adjustments, facilitating more efficient hydrogen storage. Additionally, a class of emerging materials known as covalent organic frameworks (COFs) shares structural features akin to MOFs. However, owing to their predictable synthesis methods and flexible structural design, COFs are also regarded as possessing the potential for physical adsorption hydrogen storage applications. In summary, physical adsorbent materials play a crucial role in hydrogen storage, with activated carbon, carbon nanotubes, MOFs, and COFs continuously evolving and being optimized to provide further avenues for efficient and safe hydrogen storage and utilization [49,50]. Nonetheless, the low hydrogen storage capacity (<2 wt.%) of physical adsorbent materials at ambient temperature and pressure significantly limits their practicality in solid-state hydrogen storage systems.

**Figure 2:**
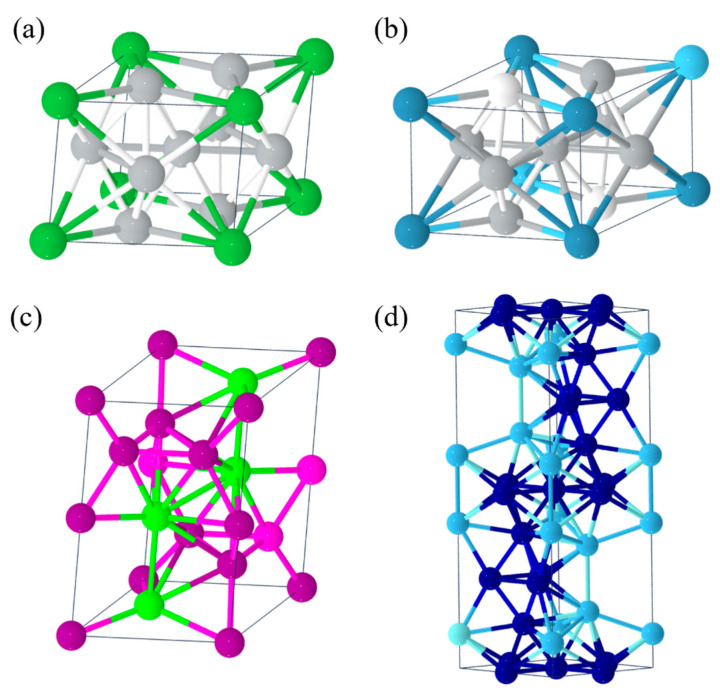
Crystal structure of (**a**) LaNi_5_, (**b**) CaNi_5_, (**c**) ZrMn_2_ and (**d**) TiCr_2._

**Figure 3:**
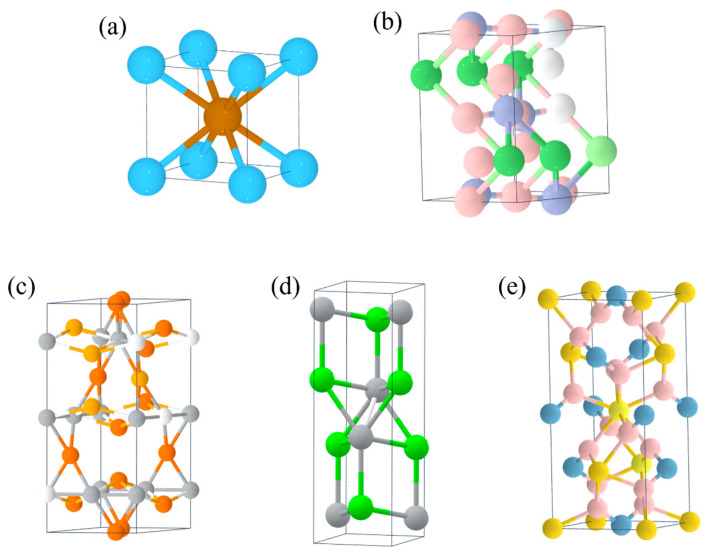
Crystal structure of (**a**) TiFe, (**b**) ZrNi, (**c**) Mg_2_Ni, (**d**) NaAlH_4_ and (**e**) LiNH_2_.

At present, the main types of hydrogen storage materials with practical application value are as follows:

(1) AB_5_-type metal hydrides such as LaNi_5_ [51], CaNi_5_ [52] (Figure 2a,b), etc., with a mass hydrogen storage density of 1.4–1.8 wt.% and a volumetric hydrogen storage density of up to 115 kg/m^3^. The dehydrogenation plateau pressure is 0.1–0.3 MPa, and the dehydrogenation temperature is 25–100 °C. These materials have good activation properties and moderate absorption/desorption pressure, but they are expensive (USD >1428.6/ton) and are mainly used as negative electrodes for nickel–metal hydride batteries.
(2) AB_2_-type metal hydrides such as ZrMn_2_ [53], TiCr_2_ [54] (Figure 2c,d), etc., with a mass hydrogen storage density of 1.8–2.2 wt.% and a volumetric hydrogen storage density of up to 130 kg/m^3^. The dehydrogenation plateau pressure is 0.1–1 MPa, and the dehydrogenation temperature is 25–150 °C. These materials have a high hydrogen storage capacity and good cyclic stability, but they are costly and difficult to activate. They are currently mainly used for large-scale stationary hydrogen storage.
(3) AB-type metal hydrides such as TiFe [55], ZrNi [56] (Figure 3a,b), etc., with a mass hydrogen storage density of 1.5–2.0 wt.% and a volumetric hydrogen storage density of 105–115 kg/m^3^. The dehydrogenation pressure is 1–3 MPa, and the dehydrogenation temperature is 25–100 °C. These materials have a moderate hydrogen storage capacity and good cycling performance, but they require high hydrogen purity (>99.99%) and are mainly used for hydrogen purification and separation.
(4) A_2_B-type metal hydrides such as Mg_2_Ni [57] (Figure 3c), etc., with a high mass hydrogen storage density of 3–5 wt.% and a volumetric hydrogen storage density of up to 110 kg/m^3^. The dehydrogenation pressure is 0.1–0.3 MPa, but the dehydrogenation temperature is high (250–400 °C). The notable feature of these materials is their high mass hydrogen storage density, but their dehydrogenation kinetics are poor and they are prone to pulverization, so their practical performance needs to be improved.
(5) Complex hydrides such as NaAlH_4_ [58], LiNH_2_ [59] (Figure 3d,e), etc., with a mass hydrogen storage density of up to 5–10 wt.%, but a dehydrogenation temperature generally above 150 °C. The advantage of these materials is their high hydrogen storage capacity, but the dehydrogenation process is often accompanied by by-products (such as NH_3_), causing the reversible hydrogen storage capacity to decrease rapidly. They are currently still in the research stage.

Table 3 gives the performance parameters of several representative solid-state hydrogen storage materials. It can be seen that each type of material has advantages and disadvantages in terms of their hydrogen storage capacity, thermodynamics, kinetics, etc. For example, the AB_5_ type has a moderate hydrogen storage capacity but a high cost, the A_2_B type has a high hydrogen storage capacity but a high dehydrogenation temperature, and complexes have an even higher hydrogen storage capacity but are prone to decomposition during dehydrogenation [60,61,62,63]. Therefore, developing new hydrogen storage materials with high capacity, fast kinetics, and a long cycle life is the focus of current basic research on solid-state hydrogen storage.

As mentioned earlier, solid-state hydrogen storage uses chemical adsorption, physical adsorption, and other interactions to reversibly store hydrogen in solid materials. Compared with gaseous and liquid hydrogen storage, its advantages mainly include the following:

(1) High energy density. The volumetric hydrogen storage density of metal hydrides can reach 100–130 kg/m^3^, which is three times that of 70 MPa gaseous hydrogen and two times that of liquid hydrogen.
(2) Good safety. Hydrogen storage materials can exist stably at room temperature and atmospheric pressure, and even if damaged, they will not cause a large amount of hydrogen leakage.
(3) Simple system. Solid-state hydrogen storage does not require gas cylinders or compressors, let alone ultra-low-temperature insulation devices, greatly reducing the complexity of the system.
(4) Good economy. Atmospheric storage can significantly reduce equipment costs, and there is no liquefaction power consumption, with operating costs that are only 1/3 of liquid hydrogen.

For solid-state hydrogen storage, the equipment costs include solid hydrogen containers and related systems for hydrogen adsorption/desorption, which account for approximately 50% of the total cost. Operational costs account for about 10–20%, with the main portion being the energy consumption generated during the hydrogen adsorption/release process. Unlike the other two hydrogen storage technologies, the safety and regulatory costs are relatively low for solid-state hydrogen, with more expenditures allocated to research and development costs, accounting for approximately 20–30% of the total cost. According to estimates, after the commercialization of solid-state hydrogen technology, the transportation price ranges approximately from USD 1.6 to USD 2.3 per kilogram for transport distances of 50–500 km.

Of course, the technological maturity of solid-state hydrogen storage is not as good as gaseous and liquid hydrogen storage, and it still faces many challenges in hydrogen storage materials, thermal management, system integration, etc. For example, the cost of hydrogen storage alloys is high (AB_5_ type: USD >14,285.7/ton; AB_2_ type: USD 0.7–1.1 million/ton), while magnesium-based materials with a higher hydrogen storage capacity have problems such as poor kinetics, easy pulverization, and a high dehydrogenation temperature. Therefore, developing high-performance and long-life hydrogen storage materials is a top priority.

## 3. Advancements in Solid-State Hydrogen Storage Systems

### 3.1. Solid-State Hydrogen Storage System Architecture

**Figure 4:**
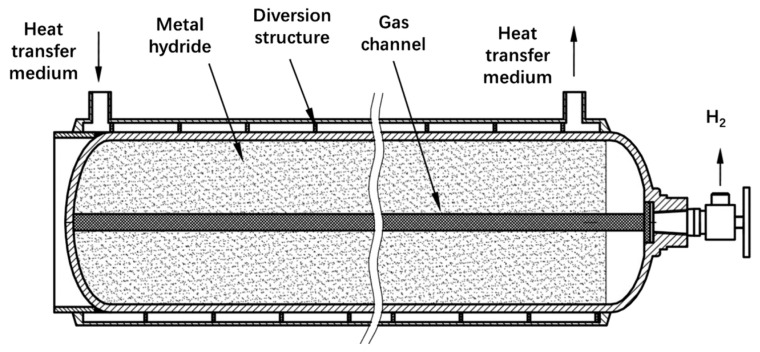
Solid-state hydrogen storage device [64].

A complete solid-state hydrogen storage and supply system mainly consists of the following parts (Figure 4) [64]: (1) Hydrogen storage device, including a hydrogen storage container and hydrogen storage material. The container is mostly made of stainless steel or composite materials, is cylindrical in shape, and is filled with granular or block-shaped hydrogen storage material. (2) Hydrogen supply device, including a hydrogen supply valve group, piping, filters, etc. The pressure difference is used to realize the delivery and circulation of hydrogen. (3) Thermal management device, including heaters, radiators, fans, etc. Electric heating or hydrogen supply tail gas heating is used to provide the heat required for dehydrogenation, while fins or forced air cooling are used to remove the heat of the exothermic reaction. (4) Monitoring device, including temperature, pressure, flow, leakage and other sensors, as well as controllers, human–machine interfaces, etc. It can realize parameter monitoring and feedback control.

The key component is the hydrogen storage container. In order to improve the heat and mass transfer efficiency and reduce the system volume, the design of the hydrogen storage container needs to comprehensively consider the following factors:

(1) Select materials with good thermal and hydrogen conductivity, such as metal materials such as stainless steel and aluminum alloy, or light-weight composite materials such as carbon fiber and glass fiber.
(2) Optimize the size and shape parameters of the container to balance the hydrogen storage capacity, heat dissipation, and layout convenience. Commonly used shapes include cylinders, annuli, corrugated pipe, etc.
(3) Set up porous baffles or fillers in the container, which can fix the hydrogen storage material particles, prevent stress concentration, and promote hydrogen diffusion and heat transfer.
(4) Set up the fin and other enhanced heat transfer elements on the outside of the container to increase the heat transfer area. The fins can be arranged axially, radially or spirally.
(5) When necessary, set up a skeleton, porous matrix, etc., in the container to guide hydrogen transport by capillary force and provide mechanical support.

The hydrogen storage material is the core of solid-state hydrogen storage, and its performance directly determines the system’s hydrogen storage capacity, kinetics, cycle life and other indicators. From a materials science perspective, the main factors affecting the hydrogen storage performance are as follows: composition, crystal structure, particle size, specific surface area, doping elements, etc. At present, the hydrogen storage materials for engineering applications mainly include the following:

(1) AB_5_-type metal hydrides, such as LaNi_5_ [65,66]. Their hydrogen absorption platform pressure is moderate (0.1–0.3 MPa), and they can release hydrogen at room temperature, but they are expensive and the cost is too high for large-scale hydrogen storage. They are mostly used in small devices such as nickel–metal hydride batteries.
(2) TiFe-based metal hydrides [67,68]. Their advantages are good cycle stability and a long service life, but they require high hydrogen purity and a high dehydrogenation pressure (1–3 MPa), and are generally used in the field of hydrogen purification.
(3) Low-temperature AB_2_-type metal hydrides, such as Ti-Zr-V series [69,70,71]. Their hydrogen storage capacity is relatively high (≥2 wt.%), their dehydrogenation pressure is moderate (0.1–1 MPa), and their price is relatively cheap, but they are difficult to activate and require high-temperature treatment above 400 °C. They are currently mainly used in large-scale stationary hydrogen storage devices.
(4) High-temperature Mg-based metal hydrides, such as Mg_2_Ni [72]. Their biggest feature are their high mass hydrogen storage density (≥3 wt.%), but their dehydrogenation temperature is also high (300–400 °C), which places high requirements on the heat and mass transfer process, and they are prone to pulverization, so their practical performance needs to be improved.

It can be seen that developing new hydrogen storage materials with a high capacity, room temperature dehydrogenation, and a long life is still the current research focus. In recent years, researchers have tried to use multi-element alloying, amorphous techniques, surface modification, nanosizing, doping and other means to improve material performance [73,74,75,76]. For example, introducing Ce, Co and other elements into the AB_5_ type can increase the platform pressure; doping Ti, Fe, etc., into Mg_2_Ni can reduce the dehydrogenation temperature; and coating AB_2_ powder with CNTs, graphene, etc., can improve electrical and thermal conductivity. However, the preparation cost of these new materials is relatively high, and industrialization still takes time.

### 3.2. Solid-State Hydrogen Storage Device Integration

Solid-state hydrogen storage devices can be divided into two categories according to the application scenario: on-board and stationary. The on-board type focuses on lightweighting and compactness, while the stationary type focuses on realizing a large capacity and long life. Due to their limited on-board space, hydrogen storage devices generally adopt a cylindrical shape, with a volume of several tens to several hundreds of liters. In the passenger vehicle field, many vehicle manufacturers have launched prototype or concept cars of solid-state hydrogen storage fuel cell vehicles. For example, Honda’s Clarity, Toyota’s Fine-X, GM’s Sequel, etc., all use 35 MPa AB_2_ or AB_5_-type materials, with a hydrogen storage capacity of 5–10 kg and a filling time of less than 10 min [77,78,79].

In the commercial vehicle field, Canada’s Hydro-Quebec has developed a variety of solid-state hydrogen storage fuel cell buses, equipped with an 800 L, 30 kg hydrogen storage system using vanadium-based AB_2_-type materials, which can be filled in 15 min under 35 MPa. The HY2MEGA project of Germany’s GKN company provides hydrogen storage modules for Mercedes-Benz Citaro fuel cell buses, with each module consisting of eight 50 L cylindrical containers connected in series, loaded with Ti_0.95_Zr_0.05_V_0.43_Fe_0.09_Cr_0.05_Mn_1.4_ AB_2_-type material, which can store 10 kg of hydrogen at 40 °C/4 MPa and support a hydrogen supply rate of 350 kg/day [80].

In terms of stationary hydrogen storage devices, Japan’s JFE company uses multiple 250 L hydrogen storage tanks in parallel, using La_0.6_Y_0.4_Ni_4.5_Co_0.4_Al_0.3_ AB_5_-type material, which can store 100 kg of hydrogen at room temperature/0.9 MPa. The Mg_2_Ni-based stationary hydrogen storage device developed by Canada’s Hydro-Quebec can achieve a hydrogen supply rate of 5 kg/h at the 200 kg level. The AIST alloy hydrogen storage tank developed by Japan’s Aichi Institute of Technology adopts an annular structure and can store about 700 g of hydrogen at 120 °C/1 MPa.

It is worth mentioning that in recent years, China has also shown its potential in the field of solid-state hydrogen storage. Xiamen University has developed a kW-level MgH_2_–NaBH_4_ composite hydrogen storage device, using a double-layer hydrogen storage tank structure, with the inner layer for NaBH_4_ hydrolysis for the production of hydrogen and the outer layer for MgH_2_ hydrogen storage and heating, which can release 7.8 wt.% of hydrogen at 150 °C. Tsinghua University has developed an AB_5_–Mg_2_Ni composite hydrogen storage system using heat pipe technology to enhance heat transfer, with a dehydrogenation capacity of 420 g at 150 °C. Sichuan University has cooperated with the National Pipeline Network Group to build a 500 kg level AB_2_–MOF composite hydrogen storage and supply system, with AB_2_ providing fast dehydrogenation at room temperature and the MOF undertaking high-capacity hydrogen storage, achieving dynamic balance.

### 3.3. Key Technologies for Solid-State Hydrogen Storage

In terms of material development, alloying is currently the main means of improving the thermodynamic properties of hydrogen storage [81,82,83,84]. Studies have shown that partially replacing La in LaNi_5_ with Mm (a mixture of La, Ce, Pr, Nd) can increase the equilibrium pressure by nearly one-fold; doping 5 at% of In, Al, Ti, Fe, etc., into MgH_2_ can reduce the dehydrogenation temperature from 400 °C to around 250 °C.

Surface treatment is an effective way to improve hydrogen storage kinetics. For example, coating Pd on the surface of AB_5_-type powder can significantly improve its low-temperature activation performance, achieving 90% of the maximum hydrogen storage capacity at 50 °C [85]. The high-energy ball milling, plasma etching, etc., of MgH_2_ can shorten the absorption/desorption time from hours to minutes at 300 °C.

Multi-phase composites can expand the application temperature/pressure range of hydrogen storage materials [86,87,88]. Mixing AB_2_ and MOF materials in a certain proportion can achieve low-temperature (room temperature), high-capacity (2 wt.%), reversible hydrogen storage. Compositing borohydride (such as NaBH_4_) with metal hydride (such as MgH_2_) can use the hydrogen and heat generated by the decomposition of NaBH_4_ to drive the hydrogenation process of MgH_2_, greatly improving the energy density and storage/release efficiency of the hydrogen storage system [89,90,91,92].

In terms of system integration, the structural design of the hydrogen storage container is crucial. Using a combination of internally porous baffles and externally finned fins can significantly improve the heat and mass transfer effect [93,94]. Coating hydrogen-permeable membranes on the baffles and skeleton can accelerate hydrogen diffusion. Introducing multi-scale (nanometer, micrometer, millimeter) porous matrix into the hydrogen storage container can help increase the hydrogen storage capacity and volume utilization of the container [95].

Advanced thermal management technology is the key to improving the performance of solid-state hydrogen storage systems [96]. The hydrogen storage process is endothermic, and the dehydrogenation process is exothermic, so the reaction heat must be supplemented or removed in time to ensure the normal operation of the system. At present, heating is mainly achieved by electric heating, cooling is mainly achieved by the natural convection of fins, and the heat transfer efficiency is low. Introducing high-efficiency heat conduction elements such as heat pipes and heat pumps, and using hydrogen precooling and cyclic heating can greatly improve the heat transfer efficiency. In terms of heat source selection, in addition to electric heating, the waste heat of fuel cells and internal combustion engines, or renewable energy sources such as solar energy and geothermal energy, can be used to build an efficient and energy-saving solid-state hydrogen storage heating network.

Advanced detection and control technology guarantees the safe operation of solid-state hydrogen storage systems. Since the hydrogen storage material will undergo volume expansion and stress changes during the absorption/desorption process, it is necessary to monitor the deformation and strain parameters of the hydrogen storage container online and adjust the control strategy in time. At the same time, it is also necessary to monitor the pressure, temperature, flow rate, purity, etc., of the system. Once an overrun or leakage occurs, the hydrogen supply should be cut off and protection should be activated in time. At present, the online monitoring technology for conventional parameters such as pressure and temperature is relatively mature, but the real-time sensing of the hydrogen concentration and material deformation still needs to be strengthened.

## 4. Application Scenarios and Market Prospects of Solid-State Hydrogen Storage Technology

**Figure 5:**
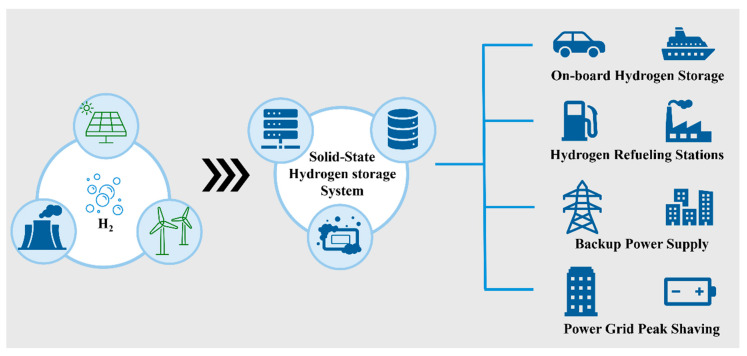
Illustration of the hydrogen industry chain.

The primary hydrogen industry chain is depicted in Figure 5 below. Hydrogen is produced through various methods, including the electrolysis of water to generate electricity. The produced H_2_ is then stored using solid-state hydrogen storage systems. Subsequently, H_2_ can be utilized in four main areas: (1) on-board hydrogen storage, (2) hydrogen refueling stations, (3) backup power supply and (4) power grid peak shaving.

### 4.1. On-Board Vehicular Applications

Among all hydrogen energy applications, on-board is perhaps the scenario that can best utilize the advantages of solid-state hydrogen storage [97]. Traditional vehicle hydrogen storage systems use 70 MPa high-pressure hydrogen storage cylinders, which have problems such as a large volume (up to 200–300 L), heavy weight (up to 100–200 kg), and high cost (USD 4285.7–5714.3 per kg of hydrogen), seriously restricting the cruising range and economy of fuel cell vehicles [98,99,100,101]. Using solid-state hydrogen storage is expected to reduce the system volume by 30–50%, reduce the weight by 30–40%, and reduce the cost by 20–30% while ensuring safety [102,103,104,105,106].

Take passenger vehicles as an example. At present, the cruising range of mainstream fuel cell cars is around 500 km, with a single vehicle carrying 5–6 kg of hydrogen and using 70 MPa high-pressure hydrogen storage cylinders [107,108,109,110]. If Mg_2_Ni-based materials are used instead, a hydrogen storage capacity of 7 wt.% (mass percentage) can be provided at 250–300 °C. Based on an estimate of 100 kg of hydrogen storage material, 7 kg of hydrogen can be stored, an increase of more than 20%. And the hydrogen storage container can be conveniently arranged in the chassis, which is conducive to optimizing the overall vehicle design.

For commercial vehicles, especially large trucks, the requirements for low-cost and long-life hydrogen storage systems are even higher. Taking the technical indicators proposed by the U.S. Department of Energy as an example, by 2025, the cost of truck hydrogen storage systems should be less than USD 8/kWh, and the cycle life should be 25,000 times longer, which is difficult for current high-pressure hydrogen storage cylinders to achieve. Using AB_2_-type hydrogen storage alloys, the reversible hydrogen storage of 1.5–2 wt.% can be achieved at 100–150 °C, with a service life that is more than 10,000 times longer; the cost can also be controlled below USD 10/kWh. If coupled with high-capacity adsorption materials such as MOF, the hydrogen storage capacity is expected to be further increased by 30–50%.

Table 4 summarizes the changes in the sales and ownership of fuel cell vehicles in China and globally from 2016 to 2023. From the perspective of market capacity, according to estimates, the sales of fuel cell vehicles in China in 2020 were about 12,000 units, with an average hydrogen consumption of 1 kg/100 km per vehicle, and an annual hydrogen consumption of 1200 tons, corresponding to a market scale of about USD 140 million for hydrogen storage systems. Looking forward to 2030, with the promotion and popularization of fuel cell vehicles, it is estimated that the annual sales will reach 300,000 units. If solid-state hydrogen storage accounts for 20%, the market space will exceed USD 1.7 billion.

### 4.2. Hydrogen Refueling Stations

As the energy refueling facility for fuel cell vehicles, hydrogen refueling stations are another major potential application scenario for solid-state hydrogen storage. Unlike traditional liquid fuels, the volume of 70 MPa high-pressure gaseous hydrogen is as high as 56 L per kilogram, so the storage and transportation of hydrogen at atmospheric pressure is very uneconomical, and the method of “high pressure + low temperature” is usually adopted for on-site liquefaction and then supplied in gaseous form [111,112,113,114,115,116]. This requires hydrogen refueling stations to be equipped with large air compressors and refrigeration equipment, with an investment of millions of US dollars. By using solid-state hydrogen storage, the investment and operation and maintenance costs of hydrogen refueling stations can be significantly reduced:

(1) Atmospheric pressure storage, no need for compressors, saving electricity;
(2) Adsorption at room temperature, no need for low-temperature insulation, simpler steel cylinders or tanks can be used;
(3) Safe and environmentally friendly, even if the hydrogen storage container is damaged, it will not cause a large amount of hydrogen leakage;
(4) Modular design, which can realize the integration of “storage–transportation–refueling”.

According to incomplete statistics, there are currently about 180 hydrogen refueling stations in operation in China, mostly performing 35 MPa refueling, with a daily refueling capacity of 500 kg per station. It is estimated that by 2025, the number of hydrogen refueling stations will exceed 1500, and if 20% adopt solid-state hydrogen storage, the market scale will reach USD 1.1 billion.

In the field of stationary hydrogen storage, in addition to hydrogen refueling stations, solid-state hydrogen storage can also be used in backup power stations, mobile base stations, etc. Take communication base stations as an example. China currently has nearly 8 million communication towers built, and operators invest more than USD 14.3 billion in diesel generator sets and battery packs every year. If a distributed power supply scheme of “photovoltaic + hydrogen storage + fuel cell” is adopted, not only can the operation and maintenance cost be reduced by more than 30%, but also low carbon usage and environmental protection can be achieved. If solid-state materials are used in the hydrogen storage link, the system efficiency can be increased by 10–20%. It is estimated that by 2025, about 5% of China’s communication base stations are expected to realize solid-state hydrogen energy storage replacement, with a market scale of about USD 710 million. Table 5 summarizes the changes in operational hydrogen refueling stations in China and globally from 2016 to 2023.

### 4.3. Backup Power Supply

Combined with fuel cells, solid-state hydrogen storage can be widely used in industrial parks, data centers, hospitals and other scenarios with high power supply reliability requirements. Take data centers as an example. Traditional diesel generator sets have a long start-up time (≥10 s), high noise (≥95 dB), and excessive exhaust emissions, while fuel cells can achieve instant response (≤3 s), silent operation (≤70 dB), and zero emissions, and can complement the grid and photovoltaics, extending the continuous power supply time from several hours to several days or even weeks [117,118,119,120,121].

The choice of hydrogen storage method has a great impact on system performance. At present, 35 MPa high-pressure gas cylinders or long tube trailers are mostly used for hydrogen supply, which have problems such as a large footprint and frequent refueling. Using solid-state hydrogen storage, the hydrogen storage capacity per unit volume can be increased by 2–3 times, thereby greatly reducing the hydrogen storage space and the number of replacements. And solid-state materials can be reused multiple times with a service life of more than 20 years. If fully promoted, by 2025, the market scale of solid-state hydrogen storage in data centers is expected to exceed USD 285.7 million.

In addition to data centers, backup power supplies for industrial parks are also an important entry point for solid-state hydrogen storage. At present, hundreds of chemical industrial parks in China have built regional gas turbine power plants, but due to the limitation of natural gas pipeline coverage, these power plants mostly use diesel engines as emergency power supplies, which have problems such as high pollution and low efficiency. Solid-state hydrogen storage can use the industrial tail gas produced by the park to produce hydrogen; this not only consumes excess hydrogen, but also provides clean backup power, and can be switched at any time. Initially, it is estimated that if 25% of the park power plants adopt the model of “by-product hydrogen + solid-state hydrogen storage + fuel cell”, by 2025, the market scale will reach USD 214.2 million.

### 4.4. Power Grid Peak Shaving

Against the backdrop of low-carbon transition, the proportion of renewable energy will continue to increase, and the proportion of non-stable power sources such as wind power and photovoltaics connected to the grid has exceeded 20% [122,123,124,125]. This poses a severe challenge to the peak shaving capability of the power grid. At present, thermal power peak shaving and pumped storage meet the demand with difficulty, and there is an urgent need to develop more flexible and efficient new energy storage technologies [126,127,128,129]. Hydrogen energy storage happens to meet this demand. Using electrolyzed water to produce hydrogen, excess wind and solar power can be converted into hydrogen and stored; using fuel cells to generate electricity, hydrogen can be converted back into electrical energy and released when power is in short supply, thereby achieving “peak shaving and valley filling” [130,131,132,133]. Compared with other energy storage methods, hydrogen energy storage has the advantages of large power (100 MW level), a high capacity (100 MWh level), and long life (>20 years).

Solid-state hydrogen storage can clear obstacles for the large-scale application of hydrogen energy storage power stations. Traditional liquid hydrogen tanks are expensive (USD ≥ 2857.1/kg) and have evaporation losses (1% per day). Solid-state hydrogen storage materials can be used at room temperature without the need for cryogenic treatment and high-pressure containers, thus greatly reducing system costs. It is estimated that for a 100 MWh solid-state hydrogen storage power station using magnesium-based materials, the cost per kWh can be as low as USD 0.21, which is equivalent to pumped storage. Considering the modular design advantages of solid-state hydrogen storage, it can also be deployed near wind and solar power generation areas to reduce power transmission losses.

At present, China has launched several solid-state hydrogen storage demonstration power station projects. For example, the Qinghai Golmud photovoltaic hydrogen production project is equipped with a 5-ton-level Mg_2_Ni hydrogen storage device with a daily hydrogen production of 1 ton; the Inner Mongolia Ulanqab wind power hydrogen production project uses AB_2_–MOF composite materials with an annual hydrogen production of 2 million standard cubic meters; and the Zhangjiakou renewable energy hydrogen storage project uses the MgH_2_-LiBH_4_ system with a designed hydrogen storage scale of 100 tons, which can realize a peak shaving and valley filling of 100 MW of wind and solar power. Looking forward to 2030, with the rapid growth of renewable energy installed capacity, it is estimated that China will add 50–80 GW of hydrogen energy storage power station installed capacity. If 20% adopt solid-state hydrogen storage, the market scale is expected to reach USD 8.5–14.2 billion.

## 5. Challenges and Countermeasures for the Industrialization of Solid-State Hydrogen Storage

### 5.1. Key Materials and Equipment

Although basic research on solid-state hydrogen storage has made great progress, there are still many challenges to truly realizing industrial application. The biggest bottleneck currently restricting industrialization is hydrogen storage materials. The main problems are as follows:

(1) High preparation cost. Take AB_5_-type hydrogen storage alloys as an example. The high-purity powder materials prepared by vacuum induction melting + hydrogen decrepitation treatment have a price as high as USD 11,428–14,285/ton, which is more than four times that of ordinary nickel–metal hydride battery negative electrode materials. Magnesium-based materials have similar problems. Due to the need for an oxygen-free and moisture-free environment in the preparation process, the production cost remains high and it is difficult to achieve large-scale application.
(2) Difficult batch preparation. From laboratory small-scale to industrial scale-up, hydrogen storage materials often face many process bottlenecks. For example, the AB_2_ type has difficulty obtaining a uniform multi-element solid solution, and Mg_2_Ni has difficultly avoiding pulverization and sintering. As a result, the performance of materials produced in batches is difficult to guarantee, with rapid decay and a short service life.
(3) Lack of equipment. A high preparation temperature (≥1000 °C), strict atmosphere requirements (high vacuum, high purity hydrogen), and long cycle (several days or even weeks) place very high requirements on the parameter control and air tightness of key equipment, such as melting and hydrogenation. At present, there is still a large gap between the technical level of domestic equipment and foreign equipment, especially large vacuum induction furnaces, plasma spheroidization machines, etc., and core components mostly rely on imports.

Countermeasures and suggestions: Increase scientific research investment, focus on key core technologies such as low-cost preparation and the mass preparation of high-performance hydrogen storage materials, and accelerate the independent and localized production of key materials and core equipment. The following measures are recommended:

(1) Explore new processes for preparing AB_2_ and AB_5_-type materials in non-vacuum and atmospheric environments, such as induction suspension melting, electromagnetic stirring heat treatment, etc., and strive to reduce production costs by more than 50%.
(2) Develop new methods for the low-temperature preparation of magnesium-based hydrogen storage materials, such as reaction ball milling, plasma pyrolysis, etc., while reducing the sintering temperature (≤400 °C), suppressing material pulverization, and improving cyclic stability.
(3) Implement the “Solid-State Hydrogen Storage Material Manufacturing Equipment Innovation Project” to focus on the development of ton-level vacuum induction furnaces, rapid hydrogen absorption/desorption systems, high-efficiency powder sorting systems, etc., to break through the industrialization bottlenecks of material preparation and system integration.

### 5.2. Testing Standards and Specifications

Product testing and certification are important guarantees for the development of emerging industries. At present, there are a lack of unified testing specifications and technical standards in the field of solid-state hydrogen storage, resulting in the uneven performance of hydrogen storage materials and components produced by various enterprises, difficulties in the performance of objective evaluations, and effects on user confidence. This is mainly reflected in the following:

(1) Non-uniform material testing methods. Common testing methods such as PCT and TDS are cumbersome to operate and have poor repeatability, and the test results from different laboratories often vary greatly. And there are a lack of evaluation standards for material aging, life, etc., making it difficult to comprehensively reflect the use performance of materials.
(2) Lack of a system certification system. At present, there are no safety and reliability testing specifications for solid-state hydrogen storage systems in China, and no third-party certification bodies have been established. This not only restricts the promotion and application of solid-state hydrogen storage technology, but also sets obstacles for enterprises to participate in international market competition.

Countermeasures and suggestions: Establish a standard system for the entire solid-state hydrogen storage industry chain as soon as possible, and promulgate relevant national/industry standards for materials, components, and systems to standardize product quality and performance evaluation methods. This specifically includes the following:

(3) Establish rapid testing methods and testing specifications for the evaluation of hydrogen storage materials, formulate industry standards for key indicators such as PCT performance, thermal stability, anti-pulverization performance, and cycle life, and provide criteria for material selection.
(4) Refer to testing specifications for fuel cells, lithium batteries, etc., and formulate safety testing standards for solid-state hydrogen storage systems as soon as possible, including high and low-temperature cycling, drop, vibration, electromagnetic compatibility, etc., to provide a basis for product safety performance evaluation.
(5) Encourage scientific research institutes and testing institutions with strength to carry out the third-party certification of solid-state hydrogen storage systems, establish a scientific, standardized, and efficient testing and evaluation system, and provide a “pass” for products to enter the market.

### 5.3. Construction of Innovation Platforms

For solid-state hydrogen storage technology to go from the laboratory to industrialization, it also requires the collaborative innovation of government, industry, academia, and researchers. At present, China started late in this field, with scattered innovation resources and a lack of a collaborative innovation mechanism for the entire chain of materials, equipment, and systems, making it difficult to form a joint force for scientific and technological innovation.

Countermeasures and suggestions: With the support of national key R&D plans, we should strengthen government guidance, build a collaborative innovation platform integrating “government, industry, academia, research, finance, and intermediary”, strengthen upstream and downstream enterprise collaboration, and promote the tackling of key core technologies and the industrialization of results. Specific measures include the following:

(1) Relying on innovative carriers such as national key laboratories and manufacturing innovation centers, focusing on basic research and common technologies for solid-state hydrogen storage, and organizing and implementing the “Solid-State Hydrogen Storage Frontier Technology Research Special Project” to provide a continuous technology supply for industrial development.
(2) Actively strive for the support of the national key R&D plan, organize leading enterprises, universities, and research institutes to carry out collaborative research on various links of the industrial chain, and focus on breakthroughs in key technologies, such as the large-scale preparation of hydrogen storage materials, system integration and optimization, and batch assembly.
(3) Support the creation of “Solid-State Hydrogen Storage Industry Innovation Centers” in qualified regions, build public technology R&D platforms, pilot scale-up bases, and testing and certification centers, open up the innovation chain from materials and components to complete vehicles, and accelerate the transformation of scientific and technological achievements into real productivity.
(4) Give full play to the guiding role of venture capital and industrial investment funds, promote the gathering of various innovative resources to key links of the industrial chain by setting up “Solid-State Hydrogen Storage Special Funds” and implementing “Solicitation for Solutions”, and accelerate the industrialization process of scientific and technological achievements.
(5) Encourage local governments and industry associations to take the lead in regularly holding “Solid-State Hydrogen Storage Industry Development Forums”, inviting upstream and downstream enterprises, universities and research institutes, financial institutions, third-party service organizations, etc., to jointly “diagnose and treat” industrial development and form a joint force.

## 6. Conclusions and Outlook

Solid-state hydrogen storage is increasingly favored in the domains of new energy vehicles and distributed energy due to its inherent advantages, such as high safety, energy density, and cost-effectiveness. Although its industrialization is still in its nascent stages, breakthroughs in key technologies like hydrogen storage materials and system integration are expected to propel solid-state hydrogen storage to mainstream status, rivalling gaseous and liquid hydrogen storage within the next 10–15 years.

While China entered the solid-state hydrogen storage sector relatively late, it has been making notable progress. Scientific research institutions have achieved breakthroughs in materials such as magnesium-based materials and AB2/MOF composite materials. Concurrently, companies like Houpu Clean Energy, Xintao Energy, and Hongda Xingye are accelerating their industrial deployment. Looking ahead, it is recommended that current hydrogen energy industry trends are capitalized on, leveraging the substantial market space and robust industrial foundation of on-board hydrogen storage and hydrogen refueling stations. This strategic approach can accelerate the widespread adoption of solid-state hydrogen storage technology.

In the passenger vehicle sector, the focus should be on developing light-weight, cost-effective, and durable on-board solid-state hydrogen supply systems using magnesium-based materials. The objective is to reduce material costs to below USD 2857.1/ton and system prices to around USD 214.3/kWh within the next 5 years, surpassing high-pressure gaseous hydrogen storage. Similarly, in commercial vehicles, optimizing vanadium-based AB2-type materials is crucial to developing high-capacity (≥6 wt.%) solid-state hydrogen storage systems with fast kinetics (≤10 min). The target is to reduce system costs to below USD 71.4/kWh within the next 3 years.

In urban hydrogen refueling stations, promoting the “atmospheric pressure + room temperature” solid-state hydrogen storage process is recommended. Encouraging capable enterprises to pioneer integrated “hydrogen production–storage–refueling” stations can help reduce single-station investments to within USD 2.1 million. Moreover, exploring a new distributed energy supply model of “local hydrogen production + solid-state hydrogen storage + fuel cell” in remote areas is crucial. This involves developing stable and high-capacity magnesium-based solid-state hydrogen storage power stations, with a single-unit scale reaching megawatt-hour levels and a power generation cost lower than USD 0.07/kWh.

It is imperative to prioritize basic research, increase scientific research investments, and focus on fundamental theoretical issues in hydrogen storage material design, preparation, and application. Supported by national key R&D plans, accelerating the establishment of a robust iterative innovation mechanism is essential. Encouraging collaboration among enterprises, universities, and research institutes to form a “production–study–research–application collaborative innovation alliance” will help create a comprehensive innovation layout spanning basic research, applied development, and engineering scale-up. This approach aims to achieve independent and controllable core technologies in hydrogen storage materials and master the core competitiveness of key equipment and products.

Looking ahead, as the energy and power system reform deepens, large-scale energy storage will become integral to building a new power system. Hydrogen, as a secondary energy source with promising application prospects after electricity, plays a crucial role in efficient energy storage and utilization, impacting national energy security and carbon peak and neutrality strategies. Solid-state hydrogen storage, as an emerging technology with immense potential, is poised to make significant contributions. Emphasizing independent innovation, robust top-level design, forward planning, and focused efforts to tackle key challenges is paramount. Aligning closely with market demands, identifying focal points, promoting by sector, and establishing an open industry chain from materials and components to systems are critical strategies. Strengthening resource integration, advocating open cooperation, leveraging the leadership of leading enterprises, and fostering a collaborative innovation pattern across fields and disciplines are essential for success.

We believe that China’s solid-state hydrogen storage industry is well positioned to seize opportunities, surpass expectations, facilitate China’s transition into a hydrogen energy powerhouse, contribute to realizing the energy revolution, and offer Chinese solutions and wisdom to global climate governance.

## Figures and Tables

**Table 1:** Parameters of the four types of high-pressure gas cylinders.

Type	Type I	Type II	Type III	Type IV
Pressure (MPa)	17.5–20	20–30	30–45	45–70
Volumetric density (kg/m^3^)	10–20	20–25	30–40	30–40
Gravimetric density (wt.%)	1.6–1.8	3.6–4.2	6.2–7.0	9.0–10.0
Cycle (years)	15	15	15–20	15–20

**Table 2:** Performance metrics of liquid hydrogen storage technology.

Technologies	Cryogenic Liquid Hydrogen Storage	Liquid Organic Hydrogen Carriers (LOHC)
Volumetric density (kg/m^3^)	70	70
Gravimetric density (wt.%)	5.7	6
Pressure (MPa)	2–4	0.5–2
Temperature (°C)	−235	25
Safety	High	Middle

**Table 3:** Technical indicators of solid hydrogen storage materials.

Materials	LaNi_5_	CaNi_5_	ZrMn_2_	TiCr_2_	TiFe	ZrNi	Mg_2_Ni	NaAlH_4_	LiNH_2_
Capacity (wt.%)	1.8	1.4	1.5–2.0	2	1.8	1.6	3.6	5.6	10.5
Temperature (°C)	25	25	25–100	25–100	100	25–100	250–350	150	150
Pressure (MPa)	0.1–0.3	0.1	0.1–1	0.1–1	1–2	1–3	0.1–0.3	0.1–1	0.1–1

**Table 4:** Variations in the number of fuel cell vehicles.

Years	Sales Volume of Fuel Cell Vehicles (Units)		Stock of Fuel Cell Vehicles (Units)	
	China	World	China	World
2016	629	2755	639	2312
2017	1272	4575	1911	6475
2018	1527	5523	3438	12,900
2019	2737	10,409	6175	24,132
2020	1177	9006	7352	32,535
2021	1587	17,027	8939	49,562
2022	3367	18,631	12,306	67,488
2023	5805	14,451	12,682	-

**Table 5:** Variations in the number of hydrogen refueling stations (units).

Years	China	World
2016	10	274
2017	14	328
2018	31	369
2019	61	434
2020	128	560
2021	218	685
2022	274	815
2023	428	1089
